# Supplementary material for: Evaluation of China’s provincial digital economy development level and its coupling coordination relationship
Source: PLoS One. 2023 Jul 25;18(7):e0288614. doi: 10.1371/journal.pone.0288614 (PMC10368274; doi:10.1371/journal.pone.0288614)
Supplement: Attachment — Submitted filename: Response to Reviewers.docx [file pone.0288614.s002.docx]

1.Deleted analysis

2. Deleted Based on the National Bureau of Statistics' delineation of the industrial scope of digital economy

3. originality, gap of research, practical, academic and policy contributions of the paper have been added.

4. research questions have been added

5. This sentence has been revised.

6. References have been added

7.research gaps and filled gaps have been added

8.the short introduction has been added

9.research gaps,detailed discussion has been added

10. References have been added

11. Reasons have been given for the selection of secondary indicators

12.Reasons for multiplying by 0.99 and adding 0.01 have been added, lnPij is a common treatment in academia.

13. Descriptive statistics have been added

14. OK, if it's necessary.

15.Index evaluation has been transferred to research methods.Comparisons with other evaluation methods and comparisons with other entropy methods in the literature have been added

16. The policy contribution has been mentioned above and the limitations have been added to the conclusions and recommendations.

17. The author's name has been changed to surname in the text
