# Supplementary material for: Evaluation of China’s provincial digital economy development level and its coupling coordination relationship
Source: PLoS One. 2023 Jul 25;18(7):e0288614. doi: 10.1371/journal.pone.0288614 (PMC10368274; doi:10.1371/journal.pone.0288614)
Supplement: Attachment — Submitted filename: Response to Reviewers.docx [file pone.0288614.s003.docx]

1. 7 top journal articles have been added.

2. Except for mark 1 which has been deleted

3. The delineation of the five industry dimensions is based on the National Bureau of Statistics' delineation of the industry scope of the digital economy, which was originally mentioned in the article, but was removed in the first revision and has now been re-added.

4. Innovative statements that extend the scope to the whole country have been removed

5. "The measures of the digital economy that have been proposed in the literature fall into the following four main categories " has been merged with the next paragraph

6. The title has been shortened to "Digital economy evaluation index system construction, methods and results".

7. Relevant literature has been added to explain the selection of secondary indicators.

8. Has been amended to "research object of this paper is 30 provinces"

9. Analysis of the coupling and coordination relationship of five industrial dimensions of digital economy is the main heading of Part 5, which I think should not be deleted

10. Has been modified to Research Method

11. "coupling and coordination degree model" explanation is marked

12. Typology is type classfication, which has been unified in the text.

13. Has expanded the content of the articles by Li and Cui [16], Ge et al. [17]

14. The rating scale such as moderate is based on the results in Table 5 and also draws on the relevant divisions of Li and Cui [16] and Ge et al. [17]. Explanations have been added in the text.

15. The features section has been modified to be more obvious for comparison

16. p.25 is the conclusion of this paper, there is less existing literature of the same type and relevant comparisons have been added to the discussion.

17. A discussion section has been added.

18. Formatting changes have been made to the references.

19. Table 4 has been added for citation and elaboration.
